# The glyceraldehyde-3-phosphate dehydrogenase gene of *Moniliophthoraperniciosa*, the causal agent of witches' broom disease of *Theobroma cacao*

**DOI:** 10.1590/S1415-47572009000200024

**Published:** 2009-06-01

**Authors:** Juliana O. Lima, Jorge F. Pereira, Johana Rincones, Joan G. Barau, Elza F. Araújo, Gonçalo A. G. Pereira, Marisa V. Queiroz

**Affiliations:** 1 Departamento de Microbiologia, Universidade Federal de Viçosa, Viçosa, MGBrazil; 2 Núcleo de Biotecnologia Aplicada a Cereais de Inverno, Embrapa Trigo, Passo Fundo, RSBrazil; 3 Departamento de Genética e Evolução, Universidade Estadual de Campinas, Campinas, SPBrazil

**Keywords:** glyceraldehyde-3-phosphate dehydrogenase gene, *Moniliophthora perniciosa*, witches' broom

## Abstract

This report describes the cloning, sequence and expression analysis of the glyceraldehyde-3-phosphate dehydrogenase (*GAPDH*) gene of *Moniliophthora perniciosa*, the most important pathogen of cocoa in Brazil. Southern blot analysis revealed the presence of a single copy of the *GAPDH* gene in the *M. perniciosa* genome (*MpGAPDH*). The complete *MpGAPDH* coding sequence contained 1,461 bp with eight introns that were conserved in the *GAPDH* genes of other basidiomycete species. The *cis*-elements in the promoter region of the *MpGAPDH* gene were similar to those of other basidiomycetes. Likewise, the *MpGAPDH* gene encoded a putative 339 amino acid protein that shared significant sequence similarity with other GAPDH proteins in fungi, plants, and metazoans. Phylogenetic analyses clustered the MPGAPDH protein with other homobasidiomycete fungi of the family Tricholomataceae. Expression analysis of the *MpGAPDH* gene by real-time PCR showed that this gene was more expressed (~1.3X) in the saprotrophic stage of this hemibiotrophic plant pathogen than in the biotrophic stage when grown in cacao extracts.

Glyceraldehyde-3-phosphate dehydrogenase (GAPDH) is a key enzyme of glycolysis, although other functions have been proposed for this protein (Sirover, 1999), including a role in the pathogenesis of prokaryotic and eukaryotic pathogens (Barbosa *et al.*, 2006; Egea *et al.*, 2007). In filamentous fungi, the *GAPDH* gene is generally present as a single copy (Punt *et al.*, 1988; Harmsen *et al.*, 1992; Hirano *et al.*, 1999; Kuo *et al.*, 2004; Vastag *et al.*, 2004; Neveu *et al.*, 2007; Liao *et al.*, 2008), but some species may contain two (Harmsen *et al.*, 1992) or three (Wolff and Arnau, 2002) copies of the gene, not all of which are necessarily transcriptionally active (Harmsen *et al.*, 1992; Wolff and Arnau, 2002). The *GAPDH* gene is frequently very highly expressed, with GAPDH protein accounting for up to 5% of the total content of soluble cellular proteins in various eukaryotes (Piechaczyk *et al.*, 1984). This elevated expression raises interesting practical questions about the regulation of this gene.. The eukaryotic *GAPDH* gene is controlled by a highly active promoter that has been used to construct transformation systems for numerous fungal species (Ridder and Osiewacz, 1992; Schuren and Wessels, 1994; Cheng *et al.*, 2001; Irie *et al.*, 2001; Kuo *et al.*, 2004; Neveu *et al.*, 2007; Kuo and Huang, 2008).

The basidiomycete *Moniliophthora perniciosa* (Homobasidiomycetes, Agaricales, Tricholomataceae) is the causal agent of witches' broom disease in cacao plants (*Theobroma cacao*). This hemibiotrophic phytopathogen initially develops as a monokaryotic/biotrophic mycelium that shows slow, low density growth, during which it occupies the intercellular space of infected tissue. After this monokaryotic/biotrophic phase, the mycelium shifts to a dikaryotic/saprotrophic phase of vigorous growth in which it colonizes inter- and intracellular tissues and rapidly destroys the infected tissues (Evans, 1980; Rincones *et al.*, 2008). This disease has had a significant socio-economic effect on Brazilian cocoa production. In view of the economic importance of this phytopathogen, in 2000 a *M. perniciosa* genome sequencing consortium was established to investigate the molecular basis of this pathogen and its interaction with cacao plants (Witches' broom project). A databank of genomic sequences, based on a 6X coverage of the *M. perniciosa* genome, is currently being used for gene discovery and to support other experiments related to gene expression, physiology and histology. In this context, the creation of an efficient transformation system for *M. perniciosa* is of prime importance in order to allow functional genomic studies of this pathogen during its interaction with the host plant.

Our group recently established a heterologous transformation system for *M. perniciosa* based on the hygromycin B phosphotransferase (*hph*) gene under control of the *GAPDH* promoter of *Aspergillus nidulans* (Lima *et al.*, 2003) and *Agaricus bisporus* (Lopes *et al.*, 2008). In the present study, we cloned the *M. perniciosaGAPDH* gene (*MpGAPDH*) and analyzed its complete nucleotide sequence. The molecular organization, homology with GAPDH proteins of other fungi, and differential expression were also analyzed. The potential use of the homologous *GAPDH* promoter in future transformation experiments is discussed.

The fungal strain used was *M. perniciosa* CP02, the same strain as used in the Witches' broom project. The total DNA from this strain was used to amplify a 396 bp DNA fragment containing part of the *MpGAPDH* gene, with the primers 5' GCGAACTTTTCAATGGTGGT 3' and 5' AACGAGTGCGTACCCTCAAC 3'. These primers were designed based on sequences present in the genome-sequencing consortium database (Witches' broom project) that showed sequence similarity to *GAPDH* genes of other fungi. This DNA fragment was also used as a probe to isolate recombinant phages from a genomic library of *M. perniciosa* constructed in the bacteriophage λEMBL3. DNA sequencing was done in a MegaBACE 500 sequencer (Amersham Biosciences). Subsequent analyses of DNA and protein sequences were done with the BLAST algorithm and the programs CLUSTAL W and PAUP* (version 4.0). The GenBank accession number for the *M. perniciosaGAPDH* gene sequence is DQ099333.

Southern blot analysis was done according to standard procedures (Sambrook and Russell, 2001). Total DNA (3 μg) was digested with restriction enzymes (*Bam*HI, *Pst*I and *Sac*I) and the resulting fragments then separated on a 0.7% agarose gel. Hybridizations were done at 65 °C using the Gene Images Random Prime Labeling Module and Gene Images CDP-Star Detection Module kits, according to the manufacturer's instructions (Amersham Biosciences).

The expression profile of the *MpGAPDH* gene was assessed by using real-time PCR with SYBRGreen in an ABI Prism 7500 Sequence Detection System (Applied Biosystems, Foster City, CA). Total RNA was extracted with an RNeasy plant minikit (Qiagen) from *M. perniciosa* biotrophic-like and saprotrophic mycelia grown in synthetic media containing 5% glycerol and induced with a 1% cacao-meristem extract, according to Meinhardt *et al.* (2006). Control RNA was obtained from the saprotrophic mycelium of *M. perniciosa* grown under the same conditions, but without the cacao extract. The primers 5' GGAT CTGTCGGTGCTCACTA 3' and 5' AACGTACATGG GTGCATCA 3' were used to amplify a 100 bp amplicon of the *M. perniciosa GAPDH* gene, with mp-β-actin1 (primers: 5' CCCTTCTATCGTCGGTCGT 3' and 5' AGGATA CCACGCTTGGATTG 3') and mp-60S ribosomal RNA (primers: 5' CAACTCTCTTTGAAGCGTTGC 3' and 5' CGAGGAACATGACGCAATTA 3') being used as internal housekeeping genes to normalize gene expression. Full details of the procedures used in this assay have been described elsewhere (Rincones *et al.*, 2008).

Southern hybridization of digested *M. perniciosa* genomic DNA with the 396 bp fragment of the *MpGAPDH* gene revealed a single band in each digestion (*Bam*HI, *Pst*I, and *Sac*I), indicating the presence of only one copy for this gene (data not shown). This result agrees with data for several fungal species in which a single copy of the *GAPDH* gene has been reported (Punt *et al.*, 1988; Harmsen *et al.*, 1992; Hirano *et al.*, 1999; Kuo *et al.*, 2004; Vastag *et al.*, 2004; Neveu *et al.*, 2007; Liao *et al.*, 2008). When the same 396 bp fragment of the *MpGAPDH* gene was used as a probe to screen a phage genomic library, five positive clones were isolated. A DNA fragment of ~4 kb from one of these phages was subcloned into the pBluescript II KS+ vector (Stratagene) and partially sequenced. The resulting sequence was incomplete, lacking the 3' region of the gene (approximately 700 bp). To complete the sequence, we mined the database of the Witches' broom project, from which several reads were retrieved through BLASTn comparisons and assembled *in silico* using the software Gene Projects (Carazzolle *et al.*, 2007). The coding region of the *MpGAPDH* gene consisted of 1,461 bp. We sequenced an additional 799 bp of the promoter region and 203 bp of the terminator region. The cDNA sequence of the *MpGAPDH* gene was also obtained from the Witches' broom project database. The software Gene Projects retrieved six cDNA reads that clustered to form two contigs spanning the whole transcribed region of the *MpGAPDH* gene. The presence of eight introns was determined by aligning the genomic and cDNA sequences. These introns (50, 65, 49, 54, 58, 57, 56 and 52 bp) show consensus 5'-end (GT) and 3'-end (PyAG) sequences and internal sequences (such as TAAG and CAAT) that are common to introns of filamentous fungi (Gurr *et al.*, 1987). Most introns of fungal *GAPDH* genes are located at the 5'-end. Although the position of some of these introns is highly conserved within the Basidiomycetes and Ascomycetes, only one intron position is conserved between these two groups (Harmsen *et al.*, 1992) and no intron position is conserved when compared to the Zygomycetes (Wolff and Arnau, 2002). Moreover, the average number of introns is higher in *GAPDH* genes of Basidiomycetes than of Ascomycetes. A rare intron organization has been reported for the *GAPDH* genes of *A. nidulans*, *Claviceps purpurea*, *Blumeria graminis* f. sp. *hordei* and *Pseudozyma flocculosa,* with one intron positioned outside the structural region (Punt *et al.*, 1988; Christiansen *et al.*, 1997; Neveu *et al.*, 2007). Apart from their evolutionary significance, introns from *GAPDH* genes can have other roles. Thus, for instance, one of the introns of the *GAPDH* gene has an important role in the high level of protein expression in *Phanerochaete chrysosporium* (Kuo and Huang, 2008) while other introns are important for the identification of some ectomycorrhizal basidiomycetes (Kreuzinger *et al.*, 1996).

Sequence analysis of the upstream region of the *M. perniciosa GAPDH* gene revealed the presence of several putative *cis*-elements, namely a putative TATA box (TATAAA) located at -73 bp and three putative CAAT boxes (CAAT) at -153, -106 and -86 bp that were positioned using the translation start point as a reference. The putative TATA box was followed by a long stretch of pyrimidine residues upstream of the start ATG codon, as also observed in many fungal *GAPDH* genes (Gurr *et al.*, 1987). In addition to the *cis*-elements identified in the *MpGAPDH* promoter, the region surrounding the start codon (CCCACC**ATG**GT) contained a purine (adenine) at the -3 bp position that is important for ribosome targeting (Gurr *et al.*, 1987). Additional conserved sequences described for the *GAPDH* promoter regions of the ascomycetes *A. nidulans* and *A. niger*, known as the *pgk*, *qa*, *gpd*, and *qut* boxes (Punt *et al.*, 1990) were not found in the *MpGAPDH* gene, in agreement with the observations in other basidiomycetes (Harmsen *et al.*, 1992; Kuo *et al.*, 2004) and one zygomycete (Wolff and Arnau, 2002).

This lack of similarity among the *GAPDH* promoter regions of different fungal phyla may explain why the regulatory region of an ascomycete *GAPDH* gene is usually poorly recognized by the transcriptional machinery of basidiomycete fungi (Harmsen *et al.*, 1992). For instance, the *A. nidulans GAPDH* promoter used in the transformation system described for *M. perniciosa* appears to be active at a low rate since all of the hygromycin-resistant transformants analyzed showed a high copy number of the pAN7-1 vector (Lima *et al.*, 2003). We expect that the future use of the homologous *GAPDH* promoter described here will increase the efficiency of transformation and the number of single vector integrations in *M. perniciosa*, as has been described in a number of other fungal species (Ridder and Osiewacz, 1992; Schuren and Wessels, 1994; Cheng *et al.*, 2001; Irie *et al.*, 2001). The termination codon TAA, the most frequently found codon in filamentous fungi, was also present in the *M. perniciosa GAPDH* gene. The 3' untranslated sequence was characterized as AT-rich, although the consensus sequence (AATAAA) described by Gurr *et al.* (1987) was not identified.

The *M. perniciosa GAPDH* gene encoded a 339 amino acid protein (MPGAPDH) with a calculated molecular mass of 36.9 kDa. The *M. perniciosa* GAPDH protein showed high sequence similarity to GAPDH proteins of other organisms: 81.7% identity with the GAPDH of *Armillariella tabescens*, 78.5% with that of *Lentinus edodes*, 77.3% with *P. chrysosporium*, 76.1% with *A. bisporus* and 70.3% with *Ustilago maydis*. The *M. perniciosa* GAPDH protein was also highly similar to the GAPDH protein of some plants and animals. The cysteine residue that defines the protein's catalytic site (binding site for glyceraldehyde-3-phosphate) was located at position 150 in the MPGAPDH protein (Figure 1), the same position found in the basidiomycete *A. bisporus* and the zygomycete *Rhizomucor miehei* (Vastag *et al.*, 2004). In the basidiomycetes *Schizophyllum commune*, *P. chrysosporium*, and *L. edodes*, this cysteine occurs at position 151 (Harmsen *et al.*, 1992; Hirano *et al.*, 1999). An evolutionary tree based on the GAPDH amino acid sequence of 42 species (Figure 2) showed separation of the basidiomycetes, zygomycetes and ascomycetes, and confirmed that MPGAPDH clustered with other homobasidiomycetes of the family Tricholomataceae. Furthermore, the yeast-like ascomycetes (Saccharomycetes) were separated from the filamentous ascomycetes and the basidiomycetes were divided into homo- and heterobasidiomycete species, except for *Thanatephorus cucumeris*, which clustered with the heterobasidiomycetes species analyzed. These findings agreed with the trees previously described by others (Wolff and Arnau, 2002; Neveu *et al.*, 2007).

Real-time PCR analysis revealed that the *MpGAPDH* gene was differentially expressed in biotrophic-like (-1.528 fold-change) and saprotrophic (-1.161 fold-change) mycelia grown in cacao extract (~32% lower expression in the former compared to the latter). This gene was also repressed in both types of mycelia when compared to control saprotrophic mycelia grown in glycerol. This finding agreed with results for *M. perniciosa* cultured *in vitro* and with published DNA microarray data (Rincones *et al.*, 2008)*.* Biotrophic mycelia of *M. perniciosa* are only found in infected cacao tissue during the “green broom” stage of the disease and are very difficult to maintain *in vitro* (Evans, 1980). Spores of this homothallic species grown on typical glucose-containing culture media rapidly undergo dikaryotization by selfing to produce the saprotrophic phase, unless the media contains cocoa or potato calluses (Evans, 1980; Griffith and Hedger, 1994) or glycerol is used as the sole carbon source (Meinhardt *et al.*, 2006). Since GAPDH is one of the key enzymes of glycolysis and the biotrophic phase is unable to grow on glucose as a sole carbon source, the repression of this gene in biotrophic mycelia would be expected. Consequently, the differential regulation of *MpGAPDH* gene expression described here must be considered when its promoter is used to construct a transformation vector.

**Figure 1:**
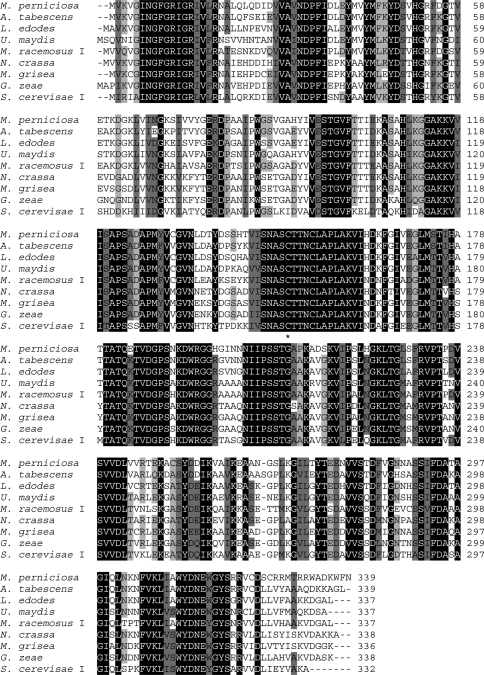
Multiple alignment of the deduced amino acid sequence of *M. perniciosa* GAPDH and other fungal GAPDH proteins (see Figure 2 for GenBank accession numbers). Gaps (-) were introduced to maximize the alignment. The level of amino acid similarity among the sequences is indicated by different shades of gray. A conserved cysteine residue (C^150^ in *M. perniciosa*) that is important for the binding of glyceraldehyde-3-phosphate in the catalytic site is indicated with an asterisk (*).

**Figure 2:**
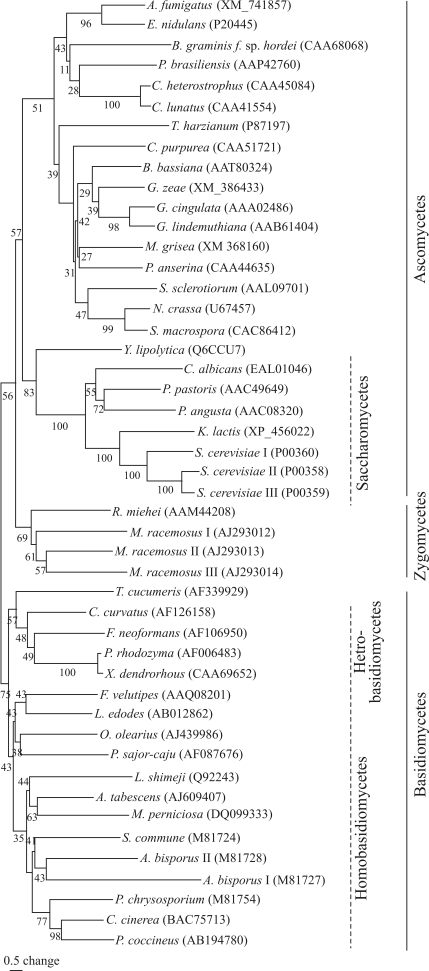
An unrooted phylogenetic tree showing the relationship between GAPDH proteins from *M. perniciosa* and other fungi (GenBank accession numbers are indicated in parentheses). The tree was built using the neighbor-joining method (Saitou and Nei, 1987). The numbers above or below each horizontal line correspond to the frequency of each branch in 1000 bootstraps.
